# Synthesis of Functional Silver Nanoparticles and Microparticles with Modifiers and Evaluation of Their Antimicrobial, Anticancer, and Antioxidant Activity

**DOI:** 10.3390/jfb11040076

**Published:** 2020-10-23

**Authors:** Erum Dilshad, Mehmoona Bibi, Nadeem Ahmed Sheikh, Khairul Fikri Tamrin, Qaisar Mansoor, Qaisar Maqbool, Muhammad Nawaz

**Affiliations:** 1 Department of Bioinformatics and Biosciences, Faculty of Health and Life Sciences, Capital University of Science and Technology (CUST), Islamabad 44000, Pakistan; mehmoona.bibi2@gmail.com; 2 Department of Mechanical Engineering, Faculty of Engineering and Technology, International Islamic University, Islamabad 44000, Pakistan; nadeemahmed@iiu.edu.pk; 3 Department of Mechanical and Manufacturing Engineering, Faculty of Engineering, University Malaysia Sarawak (UNIMAS), Kota Samarahan 94300, Sarawak, Malaysia; tkfikri@unimas.my; 4 Institute of Biomedical and Genetic Engineering (IBGE), Islamabad 44000, Pakistan; q.mansoor@ibge.edu.pk; 5 National Institute of Vacuum Science and Technology (NINVAST), Islamabad 44000, Pakistan; qaisar.vu@gmail.com; 6 Department of Rheumatology and Inflammation Research, Institute of Medicine, Sahlgrenska Academy, University of Gothenburg, 413 46 Gothenburg, Sweden

**Keywords:** silver nanoparticles, silver microparticles, functional nanomaterials, anticancer, antimicrobial, antioxidant, cytotoxicity, therapeutic efficacy

## Abstract

An accumulating body of evidence reports the synthesis and biomedical applications of silver nanoparticles. However, the studies regarding the use of maleic acid and citric acid in the synthesis of nano-sized silver particles (AgNPs) and micro-sized silver particles (AgMPs) as well as their antibacterial, antifungal, and anticancer activities have not been reported. In the current study, we synthesized AgNPs and AgMPs using maleic acid and citric acid as capping agents and have characterized them by UV-Vis, energy-dispersive X-Ray spectroscopy (EDS), X-Ray diffraction (XRD), and scanning electron microscope (SEM) analysis. The capped silver particles were examined for their antimicrobial activity and cytotoxicity against bacteria, fungi, and brine shrimp. Additionally, the anticancer activity of these particles was tested against human breast and liver cancer cell lines. The free radical scavenging activity of capped silver particles was evaluated by 2,2-diphenyl-1-picrylhydrazyl (DPPH) assay. SEM analysis revealed a round plate-like morphology of maleic acid capped particles with an average size of 39 ± 4 nm, whereas citric acid capped particles display flower-shaped morphology with rough surfaces and an average size of 250 ± 5 nm. The uncapped AgMPs were hexagonal with 500 ± 4 nm size. EDS and XRD analysis confirmed the presence of Ag and face-centered cubic crystalline nature, respectively. Functionally, capped silver particles exhibited antibacterial activity against Gram-positive (*Staphylococcus aureus, Bacillus subtilis,* and *Micrococcus luteus*) and Gram-negative bacteria (*Salmonella setubal, Enterobacter aerogenes,* and *Agrobacterium tumefaciens*). The bactericidal activity was more active against Gram-negative bacteria with minimum inhibitory concentration (MIC) as low as 5 ppm as compared to 25 ppm for Gram-positive. Similarly, the silver particles demonstrated antifungal activity by inhibiting the growth of five fungal strains (*Mucor species, Aspergillus niger, Aspergillus flavus, Aspergillus fumigatus,* and *Fusarium solani*) up to 50% at the concentration of 500 ppm. Additionally, these particles showed substantial toxicity against brine shrimp and also significantly inhibited the proliferation of breast cancer (MCF7) and liver cancer (HePG2) cell lines (IC_50_ 8.9–18.56 µM). Uncapped AgMPs were less effective, inhibiting only the proliferation of MCF7 cells with IC_50_ 46.54 µM. Besides cytotoxicity, these particles acted as potential antioxidants, showing free radical scavenging up to 74.4% in a concentration-dependent manner. Taken together, our results showed that the modifiers affect the shape and size of silver particles and may, in part, contribute to the antimicrobial and antioxidant activity of silver particles. However, the contribution of maleic acid and citric acid in enhancing the antimicrobial, anticancer, and antioxidant potential independent of silver nano and microparticles needs to be studied further. In vivo experiments may determine the therapeutic effectiveness of silver particles capped with these modifiers.

## 1. Introduction

The need for achieving materials of preferred morphology and architecture at a nanometric scale holds great importance largely because methods used in manufacturing influence the therapeutic properties of nanoparticles strongly [1,2]. In the last few years, rigorous research has been dedicated to the fabrication of metal nanostructures due to their distinctive characteristics different from their source materials [3]. Amongst the metal nanomaterials, silver nanoparticles (AgNPs) have emerged as a playful delivery platform in the field of nanotechnology. These important materials have attained considerable interest lately because of their good conductivity [4], chemical stability [5], catalytic [6], and antimicrobial activity [7,8]. It has been shown that AgNPs exhibit strain-specific antimicrobial activity against different pathogenic bacterial strains [9], as well as against toxigenic species of fungi [10,11]. Similarly, due to their distinctive features, these nanoparticles have prognostic applications against various diseases, such as those caused by bacteria, fungi, and viruses, including tuberculosis, acquired immunodeficiency syndrome, and retinal neovascularization [12]. Lately, AgNPs are being proposed as treatment options for cancers due to the anticancer activities of these nanoparticles [13,14]. It has also been reported that their accumulation in rat tissues and organs, which may pose a risk to certain cell populations in remote sites [15], could be translocated to the blood circulation and distributed to organs [16]. However, it has also been shown that the silver content of AgNPs in rat tissues gradually decreases during the 4-month recovery period, indicating tissue clearance of the accumulated silver [17].

The resistance developed by pathogens against antibiotics and other available drugs, so-called antibiotic resistance or drug resistance, currently poses a serious health concern. The combination of AgNPs with antibiotics may have profound antibacterial effects against drug-resistant strains [18]. It has been proposed that by merging modern technologies, such as nanotechnology with the material science, i.e., utilizing the metals’ inherent antimicrobial activity, could overcome such challenges. Metal and metal oxide nanoparticles exemplify a group of materials, which are being explored with respect to their antimicrobial effects. It has been shown that the particle size was the critical factor, which exhibits the antimicrobial efficacy of the metal nanoparticles [19].

Production of novel silver nanoparticles with improved activity for medical uses is a matter of endless concern [20]. Various methods have been established to synthesize silver and silver oxide nanoparticles. For instance, the wet chemical approach and chemical reduction in silver ions in aqueous solutions with or without stabilizing agents represent the methods tailored with definite morphology and controlled biochemical properties as well as antimicrobial activities [21,22].

The stabilizing agents or modifiers play an important role in preventing the clustering of particles in the suspension. Non-ionic polymers lead to steric stabilization of the particles by forming a coating on their surface, whereas ionic polymers add further electrostatic stability to the colloids [23]. Some polymers, such as polyvinylpyrrolidone (PVP), or organic salts, such as vinyl sulfonate, exhibit a tremendous capacity to regulate the growth of inorganic crystallites, including silver. Therefore, the molecules which potentially control the particle dimensions and, consequently, their self-organization, are considered to be very important [24]. Previously, citrate capped AgNPs have been shown to exhibit inhibitory effects against planktonic and sessile bacteria and cytotoxic effect against osteoblasts [25].

Recent reports demonstrate that a photodynamic therapeutic agent conjugated with fatty acids, such as oleic acid (OA), is effective against metastatic cancer cells [26]. Of particular note, the AgNPs can be incorporated into polymers, which act as deposits of silver (Ag) ions, and show their activity upon exposure or when released into the medium [27,28]. AgNPs-containing polymer composites are currently being produced for their therapeutic use and are found to be active with antimicrobial and cytotoxic effects [22]. A limited number of studies have reported the synthesis of AgNPs with acid copolymers alone [29,30] or with fruit extracts [31]. However, studies regarding the synthesis of AgNPs capped with maleic acid and citric acid are scarce. The antibacterial, antifungal, and anticancer activities of maleic acid and citric acid capped AgNPs have not been reported.

In the current study, silver nanoparticles (AgNPs) and silver microparticles (AgMPs) were prepared and characterized using two different modifiers, such as maleic acid and citric acid, and were named citric acid-capped or uncapped AgMPs and maleic acid-capped AgNPs. The antimicrobial activity and cytotoxicity effects of AgNPs and AgMPs were evaluated against bacteria, fungi, and brine shrimp and on human breast and liver cancer cell lines. Additionally, the free radical scavenging activity of these particles was also performed. Our results show that silver particles exhibit antibacterial activity against three different species of Gram-positive and Gram-negative bacteria and also inhibited the growth of at least five different fungal strains. Their cytotoxicity was also significant against brine shrimp, breast cancer (MCF7), and liver cancer (HePG2) cell lines. Besides cytotoxicity, these nanoparticles acted as antioxidants in a dose-dependent manner. Overall, maleic acid-capped silver nanoparticles were found more effective compared to citric acid-capped and uncapped particles. These modified silver particles could be effective candidates to be explored further in the drug delivery experiments.

## 2. Materials and Methods

### 2.1. Synthesis of Silver Nanoparticles and Microparticles

All the chemicals of analytical grade, including silver nitrate (AgNO3), iron (II) sulfate heptahydrate (FeSO4·7H2O), maleic acid (C4H4O4), and citric acid (C6H8O7), were purchased from Sigma–Aldrich (Sarawak, Malaysia). To synthesize nano- and micro-sized silver particles (i.e., AgNPs and AgMPs), 0.1 M silver nitrate solution was prepared in 100 mL volume, and then iron sulfate heptahydrate was added to the silver nitrate solution to give a final concentration of 0.05 M of iron sulfate heptahydrate, as described previously [32]. Then, the maleic acid and citric acid were added to the solution in separate beakers to attain the final concentration of (0.02 M) under vigorous shaking using the magnetic stirrer at room temperature for 30 min. During this process, the change in color was observed. After centrifugation at 6000 rpm for 30 min and washing with distilled water three times, the nanoparticles were dried in the hot air oven at 60 °C, and the resultant powder was saved for further analysis.

### 2.2. Characterization of Silver Nanoparticles and Microparticles

The optical absorption features of AgNPs and AgMPs were recorded in the range of 300 to 600 nm wavelength by UV-visible spectrophotometer (UV 1602 BMS spectrophotometer, Spain). The size and morphology of the samples were investigated using scanning electron microscopy (SEM) (TESCAN SEM, VEGA 3, Warrendale, PA, USA), operating at the voltage of 20 kV (maximum) with the counting frequency of 2368 cps (maximum). Chemical composition was confirmed by energy-dispersive X-ray (EDS) (Oxford Instruments, Concord, MA, USA) coupled with the SEM as plugin hardware. Magnified micrographs were taken up to the resolution of 10 µm in the scale bar. The samples for X-ray diffraction (XRD) analysis were performed by taking a small amount of solution and drying it on a quartz plate (XRD D8 Advance, Bruker GmbH, Germany). Furthermore, the crystallite parameters of prepared AgNPs and AgMPs were calculated using following Debye Scherrer’s relation,

D = 0.9λ/β × cosθ(1)

where D represents crystalline domain size perpendicular to the reflecting planes, λ is the wavelength of incident X-ray (1.5406 Å), β is the angular full width at half maximum in radians, and θ is the angle of diffraction also called Bragg’s angle [33].

### 2.3. Antibacterial Assay

The bactericidal property of the synthesized AgNPs and AgMPs was investigated by the disc diffusion method following the reported methodology [34]. There were seven bacterial strains used in the study, including three Gram-positive, such as *Staphylococcus aureus* (ATCC 6538), *Bacillus subtilis* (ATCC 6633), and *Micrococcus luteus* (ATCC 10240), and four Gram-negative, i.e., *Escherichia coli* (ATCC 15224)*, Salmonella setubal* (ATCC 19196), *Enterobacter aerogenes* (ATCC 13048), and *Agrobacterium tumefaciens* (AT-10). Briefly, bacterial suspension (100 μL) cultured in Luria Broth (10^5^ to 10^7^ CFU/mL of bacterial cultures) was spread on a nutrient agar plate. Then, the discs supplemented with synthesized particle suspension were placed on the labeled plates to determine the antibacterial properties at different concentrations (100, 50, 25, 5, 2.5 ppm). Further incubation was carried out at 37 °C in the incubator (WIG-105, Focused Photonics Inc., Hangzhou, China) for 24 to 48 h. The zones of inhibition were calculated after incubation and minimum inhibitory concentration (MIC) values were determined [35,36].

### 2.4. Antifungal Assay

The antifungal assay was performed by the agar tube dilution method, as reported earlier, against five fungal strains, including *Mucor* species, *Aspergillus niger, Aspergillus flavus, Aspergillus fumigatus,* and *Fusarium solani.* Briefly, 4 mL of Sabouraud dextrose agar medium was poured into test tubes with screw caps and subjected to the autoclave. After cooling to 50 °C, the media was loaded with 100 µL of particles’ stock suspension to make 500 ppm final concentration. Fungal strains were refreshed on sterile Sabouraud Dextrose Agar, and the resultant spores were inoculated into sterile normal saline, and the turbidity was adjusted to a 0.5 McFarland standard equivalent to yield 1 × 10^6^ CFU/mL. The suspension was further diluted in a 1:10 ratio in Sabouraud Dextrose Broth to give a turbidity of 5 × 10^5^ CFU/mL [37]. One hundred microliters of this suspension was added to the tubes, which were subjected to a slanted position and allowed to solidify at room temperature. Inoculation of fungal cultures was done in each tube from a 7-day-old fungal culture. Distilled water and terbinafine were added to the media to be used as negative and positive control, respectively. Incubation of the tubes was carried out at 28 °C in the incubator (WIG-105, Photonics Inc., Hangzhou, China) for 7 days and results were recorded by measuring linear growth (cm), and growth inhibition was calculated with reference to the negative control with the given formula;

(2) $$Growth\;inhibition=100\;\times \;\frac{Fungal\;growth\;in\;the\;negative\;control-fungal\;growth\;in\;the\;tube\;containing\;sample}{fungal\;growth\;in\;the\;negative\;control}$$

### 2.5. Brine Shrimp Lethality Assay for Cytotoxicity Screening

The preliminary cytotoxicity of synthesized AgNPs and AgMPs was evaluated by the brine shrimp lethality assay [38]. Artificial seawater was prepared by dissolving 34 g sea salt in one liter distilled water with continuous stirring. This was further utilized for hatching Brine shrimp (*Artemia salina*) eggs between 21 and 28 °C. After getting the hatched eggs, 5 mL of seawater was added to the labeled vials along with 100, 50, 25, 5 µM final concentrations of each test sample. After 2 days, hatched shrimp were transferred to these vials (15 shrimp per vial). The number of surviving shrimps was counted after 24 h [38]. Percentage viability was calculated by the following formula.

(3) $$Percentage\;viability=\;\frac{Control-test\;}{control}\times \;100$$

### 2.6. MTT Assay on Human Cancer Cell Lines

MTT (3-(4,5-dimethylthiazol-2-yl)-2,5-diphenyltetrazolium bromide) assay was performed to test the cytotoxic effect of synthesized AgNPs and AgMPs on breast cancer (MCF7) and liver cancer (HePG2) cell lines (ATCC: CCL-2™ and HB-8065™, respectively) according to the reported procedure [39]. Briefly, cells were cultured in DMEM supplemented with 10% FBS and 1% antibiotics (streptomycin and penicillin). All the components were purchased from Gibco (Invitrogen, Carlsbad, CA, USA). Cells at a density of ~100,000 were seeded in 96-well flat-bottom plates (Corning, USA) and incubated for 24 h at 37 °C in the incubator (Shel Lab, Cornelius, OR, USA) with 5% CO_2_ supply. After 24 h, the test material (in triplicate) was added at different concentrations (50, 40, 30, 20, 10 µM) to the wells containing the MCF7 and HePG2 cell lines. Untreated cells and the blank samples (cells with DMSO used as solvent) were used as controls. The plate was incubated again at 37 °C with 5% CO_2_ in the incubator for 24 hrs. Cell viability was then determined as follows: 10 µL MTT reagent (5mg/mL) was added to each treated sample, blank, and untreated cells and incubated at 37 °C for 3 h. After that, 100 µL of the solubilization solution (to dissolve formazan crystals) was added to the wells, and incubation was done in the dark at room temperature for 2–4 h. Finally, the absorbance of the samples was measured at 570 nm in the plate reader FLUO star Omega (BMG Labtech, Ortenberg, Germany).

### 2.7. 2,2-diphenyl-1-picrylhydrazyl (DPPH) Free Radical Scavenging Assay

The free radicals scavenging activity of AgNPs and AgMPs was evaluated using the 96-well plate, following the method reported earlier [40]. Each nanoparticle sample with a final concentration of 80, 40, 20 µM was mixed with 0.1 mM DPPH (190 µL) in a 96-well microtiter plate. Deionized water and ascorbic acid were used as negative and positive controls, respectively. The reaction was carried out in triplicate, and mixtures were incubated in the dark at 37 °C for 30 min, and then the absorbance of samples was measured at 517 nm by a microplate reader (BioTeK, Elx 800, Shoreline, WA, USA). IC_50_ was calculated using table curve software (version 4), and the free radical scavenging potential of the sample was calculated with the help of the following formula.

(4) $$Percentage\;scavenging=\;\frac{Absorb.\;\;control-\;test\;Absorb.\;}{Absorb.\;\;control}\times \;100$$

### 2.8. Statistical Analysis

All experiments and measurements were carried out in triplicate, and the values are presented as the mean ± standard error of the mean. The collected data were statistically analyzed using two-way ANOVA with the GraphPad Prism (V.5) software. The *p*-value of less than 0.05 was considered statistically significant.

## 3. Results and Discussion

### 3.1. Characterization of Silver Nanoparticles and Microparticles

#### 3.1.1. Confirmation of Synthesis

**Figure 1:**
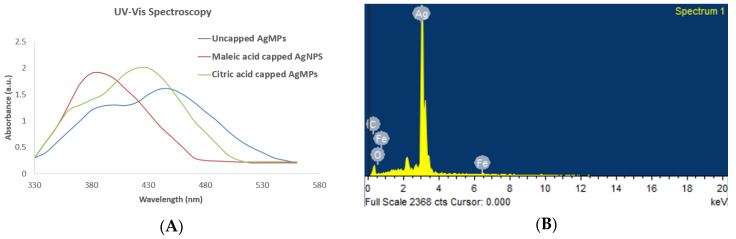
UV-Vis spectroscopy and energy-dispersive X-ray spectroscopy (EDS) analysis of synthesized silver nanoparticles and microparticles. (**A**) The spectra show the absorption peaks of maleic acid capped AgNPs (red), citric acid capped AgMPs (green), and uncapped AgMPs (blue) in the range of 350–450 nm; (**B**) The spectrum shows silver (Ag) as the major component of nanoparticles and microparticles. AgNPs: silver nanoparticles, AgMPs: silver microparticles.

First, the synthesis of silver nanoparticles (AgNPs) and silver microparticles (AgMPs) was visually confirmed. When silver nitrate and the reductant salts were mixed in particular ratios along with the modifiers, the color of the reaction turned dark grey within 10 min with continuous stirring, indicating the synthesis of silver particles. Then the synthesis was further confirmed by UV-Vis spectrum analysis, which showed a strong, broad peak at 400 nm, whereas the broadening of the peak indicates the formation of polydispersed AgNPs and AgMPs (Figure 1A). UV–Vis spectroscopy is extensively used for structural characterization of silver particles, which are known to exhibit a UV–visible absorption maximum in the range of 400–500 nm because of surface plasmon resonance excitation [34]. The peak of the citric acid-capped and uncapped AgMPs was broader than that of the maleic acid-capped AgNPs. This difference in peak could be because of their larger size range [41]. The spectrum did not contain any additional peak, thus ruling out the presence of contaminants.

Generally, for spherical shaped nanoparticles, the single peak in the UV-Vis spectra is observed [42]. The appearance of two peaks in the case of citric acid capped and uncapped AgMPs could be attributed to the anisotropic morphologies of the particles. Nanoparticles contain optical properties that are sensitive to shape size agglomeration state, concentration, and surface chemistry of nanoparticles, which makes UV-Vis spectroscopy a valuable tool for the identification and characterization of nanoparticles.

#### 3.1.2. Confirmation of Elemental Composition

Additionally, the elemental composition of the silver particles was examined by an energy-dispersive X-ray spectroscope (EDS) equipped with an SEM. The EDS profile showed a strong silver signal indicating the silver (Ag) as a major component of synthesized particles. The interpretation of the EDS spectrum was that synthesized samples are pure silver (with distinct Ag peaks) without contaminated elements. The elemental composition of particles predicted by EDS is shown in Figure 1B and the Supplementary Figure S1–S3. Peaks matching other than silver are due to the elements of the carbon-coated copper grid in the SEM measurement, and peaks corresponding to Fe maybe because of the residues of iron sulfate heptahydrate left in the suspension.

#### 3.1.3. Characterization for Structure and Size

**Figure 2:**
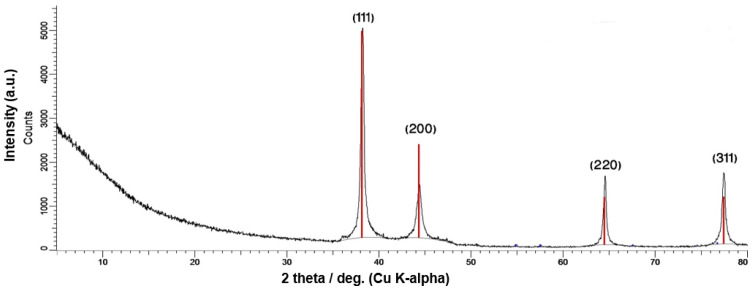
X-ray diffraction (XRD) analysis of synthesized silver nanoparticles and microparticles. The spectrum shows face-centered cubic (FCC) crystalline metallic silver nanoparticles (AgNPs) and microparticles (AgMPs). The intensity on the vertical axis is measured in counts per second (CPS), and the diffraction angle (2 theta) measured is taken along the horizontal axis. The value of wavelength in angstrom (a.u.) is also indicated.

The structural features regarding phase composition, the crystallographic orientation of AgNPs and AgMPs were further characterized by X-ray diffraction (XRD), which has long been used to outline and identify bulk materials and nanoparticles [43]. The identified peaks [38.23 (1 1 1), 44.41 (2 0 0), 64.38 (2 2 0), 77.5 (3 1 1)], revealed the crystalline nature of synthesized particles (Figure 2). The XRD outcome was found in accordance with the standard ICSD No. 98-018-0878 [40,44]. Furthermore, through Debye Scherrer’s equation and broadening of the Bragg’s peaks, it was revealed that the approximate crystallite size of maleic acid capped AgNPs are 6 nm as compared to citric acid capped AgMPs of 15 nm and uncapped AgMPs of 19 nm. These findings confirm that the synthesized AgNPs and AgMPs are made up of multiple smaller crystallite units.

#### 3.1.4. Morphology by Scanning Electron Microscopy

**Figure 3:**
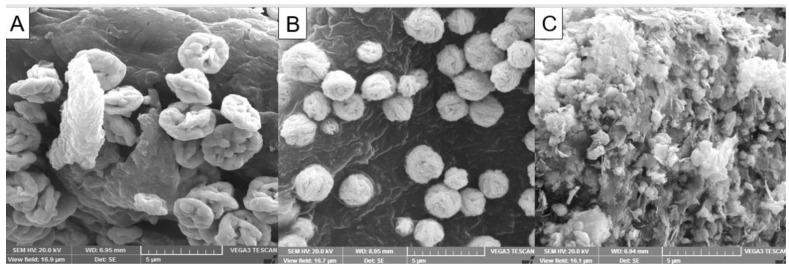
Scanning electron microscopy (SEM) micrographs of silver nanoparticles and microparticles: (**A**) uncapped silver microparticles (AgMPs); (**B**) maleic acid capped silver nanoparticles (AgNPs); and (**C**) citric acid capped silver microparticles (AgMPs). The images are presenting the shape and size of the synthesized nanoparticles. Scale bar = 5 μm.

The morphology of the synthesized AgNPs and AgMPs was examined by SEM analysis. A typical SEM micrograph of the AgNPs and AgMPs is shown in Figure 3. The uncapped AgMPs were found to be hexagonal (Figure 3A) with an average size of 500 nm ± 4. It was observed that maleic acid and citric acid controlled the shape and size of the synthesized silver particles slightly differently. When maleic acid was used as a modifier, the nanoparticles exhibited round plate-like morphology with sharp multiple facets with an average size of 39 nm ± 4 (Figure 3B). However, when citric acid was used as the modifier, the particles demonstrated a flower-like shape with rough surfaces with an average size of particles 250 nm ± 4 (Figure 3C). The ratio of the silver ions to stabilizing agents controls the size of particles produced. Hence, different factors, including the concentration of the reducing agents, metal salts, as well as time, may have played an important role in the formation of AgNPs [45]. Apart from that, stabilizing agents and modifiers also play a significant role in controlling the shape of the particles by preventing their agglomeration [23]. Modifiers in the current study include maleic acid and citric acid, which influenced the shape and size of the synthesized silver particles.

Maleic acid, being an unsaturated dibasic acid, contains one double bond, typically showing reactions of both carboxylic acids and olefins. Furthermore, there are three carboxyls and one hydroxyl in citric acid, showing typical reactions of carboxylic acids. Thus, they get absorbed on the surface of silver nanoparticles through double bonds and carboxylic acid groups. There are reports which state that silver possesses the lowest surface energy in the 111-plane, and when appropriate additives get adsorbed there, such as citrate and maleic acid [2,20], it might further decline the surface energy and could make synthesized silver plates more stable with the 111-plane as a basal plane. Both citrate and maleic acid favored the growth orientation of crystals of silver seeds. Hence, numerous plate-like silver petals accumulated and shaped secondary particles in agreement with previous reports [20,46]. Moreover, these face-centered cubic crystalline AgNPs with a (111) basal plane have previously been proven to be strong antimicrobial, cytotoxic, and antioxidant agents [36,47,48], and similar findings were observed in the current study.

### 3.2. Antibacterial Activity

**Figure 4:**
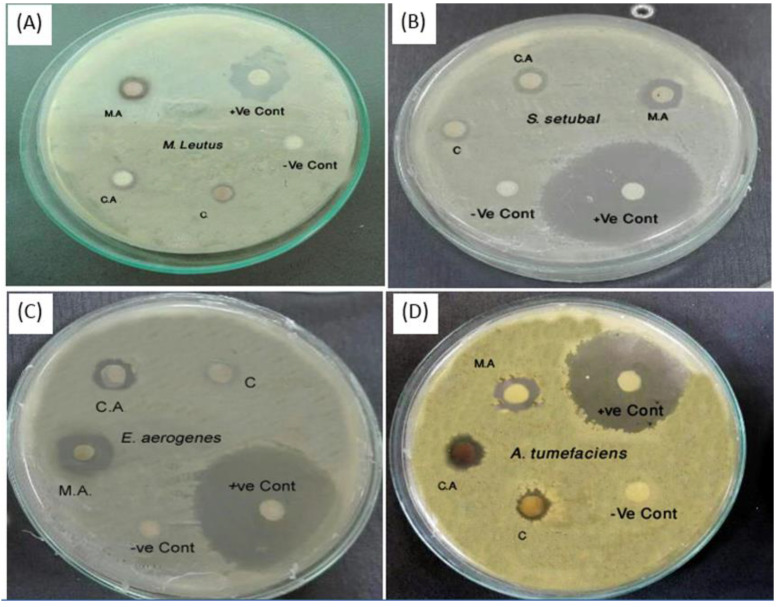
Antibacterial activity of synthesized silver nanoparticles and microparticles: Antibacterial activity of synthesized silver nanoparticles (AgNPs) and silver microparticles (AgMPs) is shown against (**A**) *M. leutus*; (**B**) *S. setubal*; (**C**) *E. aerogenes*; (**D**) *A. tumefaciens*. M.A: maleic acid capped AgNPs, C.A: citric acid capped AgMPs, C: Control or uncapped AgMPs. +ve control is streptomycin, while –ve control is distilled water.

Currently, nanoparticles are regarded as a practicable substitute for antibiotics and appear to have great potential for resolving the issues related to the development of multidrug resistance against bacteria [49]. The AgNPs are currently considered a potential candidate for the fabrication of a novel course of antimicrobials providing a novel approach to fight against a wide range of pathogens [41,50,51,52]. In the current study, synthesized AgNPs and AgMPs showed significant antibacterial activity (Figure 4). All the synthesized particles were bactericidal against studied bacterial strains, including both Gram-positive and Gram-negative.

Additionally, minimum inhibitory concentration (MIC) values were determined. Results showed that AgNPs and AgMPs appeared more effective against Gram-negative bacteria (MIC as low as 5 ppm) as compared to Gram-positive (MIC 25 ppm) (Table 1). This can be attributed to less peptidoglycan in the case of the Gram-negative bacterial cell wall [36].

Furthermore, our data show that the maleic acid capped AgNPs’ smaller size and round plat-like, yet sharp facets were more bactericidal than the citric acid capped and uncapped AgMPs with relatively larger size and rough surfaces. Previous reports have demonstrated the size- and shape-dependent activity of the AgNPs, where smaller nanoparticles appear to have more ability to infiltrate into bacteria, and they have a greater tendency to release more silver ions than larger particles to kill more pathogens [53,54,55]. It has been shown that the bactericidal activity of AgNPs is dependent on size and the dose. Previously, the citrate capped small-sized (10 nm) AgNPs at the concentration of 20 ppm showed bactericidal effect against *S. aureus* (14%), *E. coli* (53%), and *B. subtilis* (84%) [56]. When the size of the particles was further reduced to 5 nm, the bactericidal activity of AgNPs was increased. Likewise, nanoparticles made up of smaller crystallite units exhibit more antimicrobial potential, probably due to greater surface energy with higher reactivity because of condensed crystallite size. The comparative antimicrobial performance obtained in our study are well aligned with this hypothesis and also well reported in previous investigations [33]. However, in the current study, the size of the citrate capped AgMPs was comparatively greater. Therefore, they showed higher MIC values of 100 ppm for *S. aureus* and 50 ppm for *B. subtilis*. This size-dependent activity is related to the enhanced surface area to volume ratio [56].

However, the antimicrobial activity could also be attributed to organic acids themselves used in this study, i.e., maleic acid and citric acid. It has been previously reported that maleic acid and citric acid exhibit antimicrobial effects [57,58,59,60]. The antimicrobial potential of organic acid is mainly related to the reduction in microbial cell pH by ionization of undissociated acid molecules and the disruption of the transport of the substrate by changing the permeability of the cell membrane [57]. It has also been reported previously that such bactericidal activity is because of the physical interaction of AgNPs with the bacterial cell surfaces, particularly in the case of Gram-negative bacteria, where the accumulated nanoparticles were reported on bacterial surfaces [61].

However, studies have also reported that AgNPs can cause damage to the cellular membrane resulting in altered structure and thus rendering bacteria more penetrable [62,63]. This influence is highly predisposed by the nanoparticles’ morphology and concentration [62,64]. Interaction of AgNPs with *Escherichia coli* [63] enables the mechanism of action, proving that accumulated nanoparticles on cellular membrane disrupt the bilayer integrity by creating a gap. This makes the membrane more permeable, and as a result, bacterial death occurs [65]. Since enhanced permeability causes more AgNPs to enter the bacterial cell, it may cause more damage to the intracellular structures. Consequently, protein synthesis is terminated as ribosomes might have been denatured, and translation and transcription can be obstructed by targeting the bacterial cell genetic material [66,67,68].

Furthermore, it is also reported that smaller nanoparticles seem to have a superior ability to penetrate bacteria where they interact with the membrane and results in cell death. Moreover, positively charged silver ions interact with the negatively charged nucleic acid and proteins, causing structural changes and damage to the cell wall, cell membrane, and nucleic acid, as well [66,67]. This indicates that more caution will be needed in biomedical applications of silver nanoparticles.

### 3.3. Antifungal Activity

**Figure 5:**
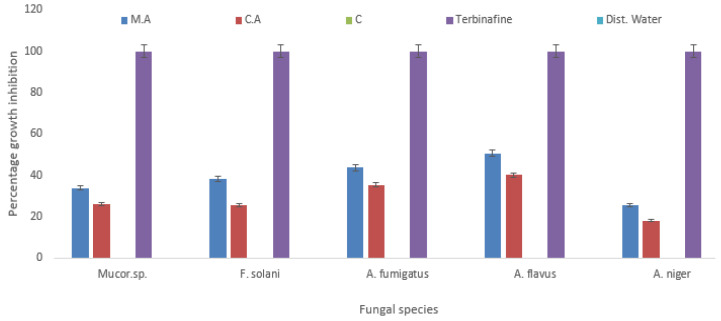
Antifungal activity of capped and uncapped silver nanoparticles and microparticles. Percentage antifungal activity of maleic acid capped silver nanoparticles (AgNPs, blue) and citric acid capped silver microparticles (AgMPs, red) is shown against different fungal species, including *Mucor species, Aspergillus niger, Aspergillus flavus, Aspergillus fumigatus,* and *Fusarium solani*. Terbinafine (purple) was used as positive control, and distilled water was used as negative control. Data are presented as the mean of triplicate readings ± standard deviation of the mean. M.A: maleic acid capped AgNPs, C.A: citric acid capped AgMPs, C: control, i.e., uncapped AgMPs.

The synthesized AgNPs and AgMPs were also found effective against five fungal strains (Figure 5). In contrast, uncapped AgMPs did not show inhibitory activity against any fungal strain. At a concentration of 500 ppm, the highest activity was observed against *Aspergillus flavus* 50.56% and 40.11% (growth inhibition) by maleic acid capped and citric acid capped particles, respectively. Whereas the least activity was observed against *Aspergillus niger* at the same concentration (500 ppm), showing 25.72% and 18.2% inhibition with maleic acid capped and citric acid capped AgNPs and AgMPs, respectively. Compared to the inhibition of bacterial species, the fungal species showed comparatively less growth inhibition. This could be because of the rigidity of the fungal cell wall, which is composed of chitin than peptidoglycan containing cell wall of bacteria [36].

Similar to their antibacterial activity, the maleic acid capped silver nanoparticles were also found to be more fungicidal, mainly because of their smaller size range and round shape with multiple sharp facets providing more surface area to interact with pathogens. This enables them to act more effectively as compared to citric acid capped silver microparticles, which were comparatively larger in size and rough flower-shaped. This is supported by previous studies [34].

Several reports support the findings that small spherical nanoparticles have better antimicrobial potential than other morphologies as these are provided with a greater surface area to volume ratio, thus proven more effective [36,42]. Large surface to volume ratio and high-atomic-density (111) aspects possibly boosted the pathogen killing proficiency [42]. It is also understood that more silver ions are released by smaller sized particles than larger ones to kill more pathogens [55]. Other reports also support these findings confirming that antimicrobial activity is reliant on the size and shape of silver particles, mainly because diverse morphologies offer different areas to intermingle with microbes and, as a consequence, end up with different competence [55,69]. The effect of capping agents in enhancing the antifungal activity cannot be ruled out as organic acids, including citric acid, have been reported to have antifungal activity [70]. 

Moreover, the interaction of AgNPs with microorganisms (bacteria, fungi, and viruses) releases silver ions (Ag^+^) that may damage the microorganism by different means; for example, they target the microbial cell wall (negatively charged) to disable cellular enzymes and interrupt membrane penetrability. Subsequently, cell disintegrates, and cell death takes place [71]. Another plausible justification could be that the silver ions also interact with enzymes and proteins’ thiol groups, which play a vital part in its antimicrobial act [67]. As nanoparticles’ resistance against pathogens has not been reported yet, thus they may have a significant benefit over traditional antimicrobial agents [72].

### 3.4. Cytotoxicity by Brine Shrimp Lethality and MTT Assay In Vitro

**Figure 6:**
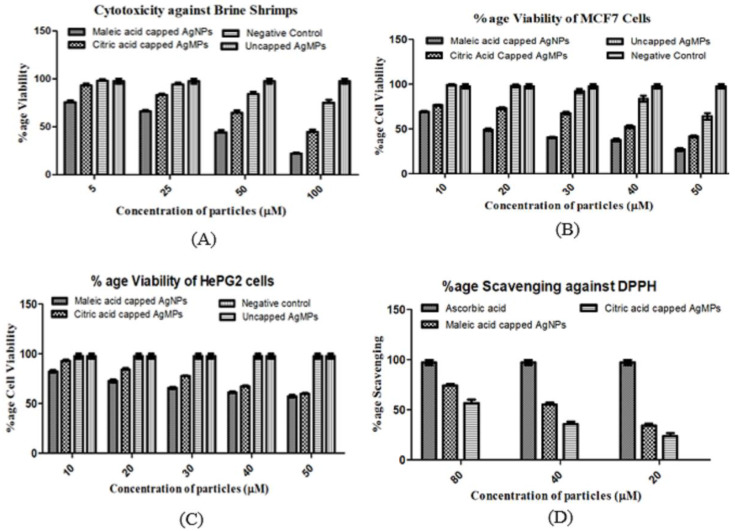
Cytotoxicity and antioxidant activity of silver nanoparticles and microparticles: (**A**) Percentage (%) cytotoxicity of silver particles against brine shrimp; (**B**) Antiproliferative activity against MCF7 cell line, (**C**) Antiproliferative activity against HePG2 cell line; (**D**) Free radical scavenging or antioxidant activity determined by 2,2-diphenyl-1-picrylhydrazyl (DPPH) assay.

Brine shrimp (*Artemia*) have extensive use in testing marine ecotoxicity and are a widely used model organism for use in toxicological assays as an acceptable alternative to the toxicity testing of mammals in the laboratory. In the current study, the cytotoxic impact of synthesized silver particles was examined on brine shrimp *nauplii* at four different concentrations, including 100, 50, 25, 5 µM. The viability of the nauplii was significantly decreased to 22% in the case of maleic acid capped, 44.3% in case of citric acid capped, and 75.3% in case of uncapped silver particles at the concentration of 100 µM (Figure 6A). The cell viability was decreased in a concentration-dependent manner, indicating that a higher concentration of silver particles is more toxic to the shrimp.

Statistically, the effect of synthesized silver particles was found to be significant (*p* < 0.001, Table 2) with IC_50_ 19.14 and 30.41 µM for maleic acid and citric acid capped silver microparticles, respectively. Whereas uncapped silver microparticles were found to be less toxic with a greater IC_50_ value of 45.1 µM. Previously, silver oxide nanoparticles synthesized using the leaf extract of *Callistemon lanceolatus* also showed dosage and time-dependent cytotoxicity against brine shrimp nauplii with an IC_50_ value of 85.32 ppm and LC_90_ value 221.8 ppm [73].

As a review for cell viability testing, an MTT assay was performed. It was observed that the viability of cultured cells reduced with an increase in the concentration of particles. At the concentration of 10 µM maleic acid capped and citric acid capped particles, the viability of breast cancer (MCF-7) cells was 69% and 76.4%, respectively (Figure 6B). However, at the concentration of 50 µM, the viability of MCF-7 cells diminished to about 25.8% and 41.3% in the presence of maleic acid and citric acid capped AgNPs and AgMPs, respectively, indicating a concentration-dependent effect. In contrast, the uncapped AgMPs at the same highest concentration, i.e., 50 µM, was found to be less toxic, which decreased viability of MCF7 cells to only 65.2%, unlike maleic acid and citric acid capped AgNPs and AgMPs, respectively, where the viability was 25.8% and 41.3%.

Similarly, the proliferation of liver cancer HePG2 cells was also downregulated in a concentration-dependent manner (Figure 6C). However, the uncapped silver particles were not found effective against this cancer cell line. Shape and size played an important role in defining the cytotoxicity of the studied particles. The impact was found highly significant (*p* < 0.001, Table 3; Table 4) with an IC_50_ value of 8.9 µM for maleic acid capped and 14.6 µM for citric acid capped and 46.54 µM for uncapped silver particles, respectively, for MCF7 cells. The IC_50_ values for HePG2 cells were found to be 9.4 to 18.5 µM in a similar respect. The concentration of the silver particles was found to be a significant factor for inhibiting the proliferation of both MCF7 and HePG2 cells. The higher the concentration of particles, the fewer viable the cells were found, and vice versa [74]. However, the capping agent might have played an important role in enhancing the antiproliferative potential of silver nano- and microparticles as maleic acid and citric acid have been reported to have anticancer properties [75,76,77].

According to ISO standards, a reduction in cell viability by more than 30% is considered to indicate cytotoxicity [78]. It has been observed that silver nanoparticles induce toxicity because of the generation of reactive oxygen species (ROS) and oxidative stress, and it has also been reported that toxicity is dependent on the size of the nanoparticles [79,80,81]. Another report described that the development of the immune response by silver nanoparticles is size-dependent. In that study, treatment of various sized silver nanoparticles (4, 20, and 70 nm) was given to human macrophages (U-937), and it was observed that nanoparticles which were the smallest in size showed the highest pro-inflammatory activity (by releasing cytokines and inducing oxidative stress) [81,82].

Apoptosis induced by silver nanoparticles of different tumor cells has already been reported in vitro and ex vivo, which possibly occurred because of the translocation of the silver within the nucleus [83,84,85,86]. Franco-Molina et al. reported that MCF-7 breast cancer cells treated with silver colloids exhibited considerably decreased dehydrogenase activity, causing NADH/NAD^+^ reduction [87]. Consequently, reduced mitochondrial membrane potential leads to cell death. Another report elaborated that phagocytized silver nanoparticles totally block the cell cycle in the S-phase and excite inflammatory signaling through ROS generation, which finally induces the secretion of tumor necrosis factor-alpha (TNF-α) [48]. Silver nanoparticles synthesized by green chemistry have been shown to exhibit anticancer activity against MCF7 and HePG2 cell lines, whereas silver oxide nanoparticles were found cytotoxic to HePG2 and Chang liver cells [48]. In the current study, the inhibition of liver cancer (HePG2) and breast cancer cells (MCF7) was caused by maleic acid- and citric acid-capped silver particles.

Moreover, it has been reported that the efficiency of silver particles can be enhanced by coating their surfaces, which not only modify their surface charge but also influence the solubility. Various reports suggest that the toxicity and fate of nanoparticles are determined by their surface coatings [88,89]. Previously different coatings have been applied to silver nanoparticles, including trisodium citrate (CT-AgNP**s**) and polyvinylpyrrolidone (PVP-AgNP**s**), which improve the physicochemical properties of silver nanoparticles, such as biocompatibility and stability, as compared to uncoated particles [90]. The modifications in the surface charge of nanoparticles through coating agents may influence their toxicity in the recipient cells. For instance, nanoparticles which have a positive charge, such as Ag+, are considered more effective drug delivery tools for anticancer drugs as compared to negatively charged ones due to their longer retention time in the bloodstream [43].

### 3.5. Antioxidant Activity

The silver particles fabricated with two different capping agents showed DPPH free radical scavenging activity (Figure 6D). The antioxidant activity of the synthesized silver nanoparticles was found to be 74.4% (maleic acid-coated nanoparticles), 55.2% (citric acid-coated silver particles) at 80 µM. It was found to be significant with *p* < 0.001 (Table 5), having a decreasing trend in a concentration-dependent manner. The low IC_50_ value of 32.03 µM was observed for smaller particles, i.e., maleic acid capped silver nanoparticles (AgNPs) as compared to larger particles, i.e., citric acid capped ones (AgMPs), which showed an IC_50_ value of 49.62 µM. However, the uncapped silver particles did not show scavenging activity.

DPPH is a stable free radical and accepts an electron or hydrogen radical to become a stable diamagnetic molecule. The antioxidants’ impact on DPPH is mainly because of their hydrogen donating activity [91]. It has been generally utilized to evaluate the radical scavenging ability of synthesized nanoparticles [40]. Kumar et al. reported good antioxidant activity of green synthesized silver nanoparticles (>78%) at 0.1mM [40].

Another report described the antioxidant effect of silver nanoparticles against DPPH, showing 75.16 ± 0.04% inhibition [47]. The antioxidant activity of silver particles in our study might be because of the capping agents (maleic acid and citric acid) adsorbed at the nanoparticles’ surface, which have been reported to possess antioxidant activities due to their ability to chelate metals, and are, therefore, classified as “preventive” or ‘’synergistic” [92]. At higher concentrations, the nanoparticles scavenged the free radicals more than at lower concentrations, possibly because more capping agents were adsorbed at the surface of nanoparticles in the case of a higher concentration, as proposed previously [74].

**Figure 7:**
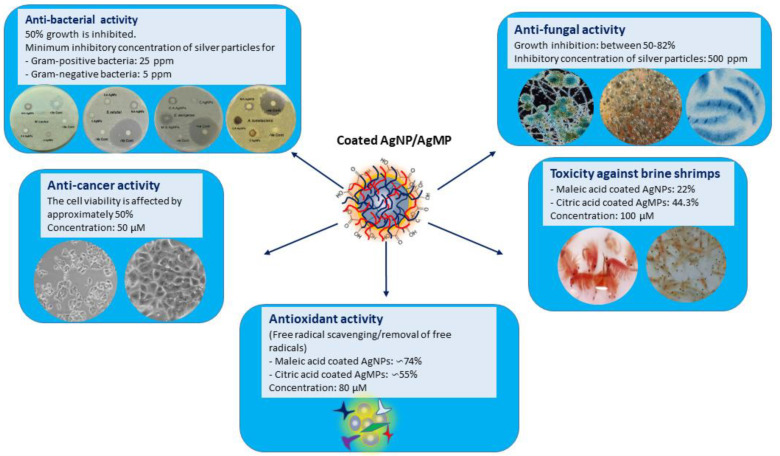
Schematic illustration representing the antibacterial, antifungal, and anticancer potential of malic acid and citric acid coated silver particles and their antioxidant activity to scavenge or remove the free radicals. AgNPs: silver nanoparticles, AgMPs: silver microparticles.

Taken together, our results showed that the modifiers act as stabilizers, and they may also be involved in enhancing the antimicrobial and anticancer potential of nanoparticles, and may, in part, contribute to the antioxidant activity of nanoparticles to scavenge or remove the free radicals (Figure 7). However, further experimentation may confirm their respective significance in the field of nanomedicine. The use of these modifiers could be a step forward to stabilize the silver nano- and microparticles and may enhance the therapeutic efficacy of silver particles capped with maleic acid and citric acid.

## 4. Conclusions

An effective and reproducible chemical reduction method for the synthesis of well-defined nano- and micro-sized silver particles has been presented, which implies that the addition of modifiers plays an important role in controlling the shape and size of the synthesized nanoparticles. The current study showed that modifiers act as stabilizers of nano- and microparticles and affect their size and shape. These modifiers may, in part, contribute to enhancing the antimicrobial and anticancer potential of silver particles and could contribute to the antioxidant activity of nanoparticles to scavenge or remove the free radicals. However, maleic acid and citric acid need to be evaluated separately for their efficacy against the studied bacterial and fungal strains and their cytotoxicity against brine shrimp and studied cancer cell lines. This study may offer an opportunity to explore the therapeutic applications of nano- and micro-sized silver particles further. However, keeping in view the accumulation of silver particles in organs or tissues, more cautions will be needed in biomedical applications of silver particles, particularly, their repeated or long-term uses.

## Figures and Tables

**Table 1:** Antibacterial activity of maleic acid capped nano-sized silver particles (AgNPs) and citric acid capped or uncapped micro-sized silver particles (AgMPs) against different bacterial strains.

AgNPsConc. (ppm)	Zone of Inhibition (cm) ± S.E																	
	Gram-positive Strains									Gram-negative Strains								
	*M. luteus*			*S. aureus*			*B. subtiles*			*A. tumefaciens*			*S. setubal*			*E. aerogenes*		
	*M.A.*	*C.A.*	*C*	*M.A*	*C.A.*	*C*	*M.A*	*C.A.*	*C*	*M.A*	*C.A*	*C*	*M.A*	*C.A*	*C*	*M.A*	*C.A*	*C*
100	1.25 ± 0.9	0.88 ± 0.5	0.4 ± 1.2	1.21 ± 0.75	0.63 ± 0.89	-	1.31 ± 0.23	0.74 ± 0.60	-	3.1 ± 1.33	2.01 ± 1.3	0.7 ± 1	1.66 ± 0.5	1.2 ± 1.3	0.54 ± 1.3	3.23 ± 0.34	2.4 ± 1.3	0.5 ± 0.9
50	0.98 ± 1.1	0.56 ± 1.21	-	0.68 ± 0.66	-	-	0.64 ± 1.15	0.52 ± 0.98	-	2.5 ± 1.55	0.85 ± 0.89	-	1.16 ± 1.1	0.66 ± 0.56	-	2.24 ± 1.3	1.4 ± 1.12	-
25	0.74 ± 1.7	0.42 ± 0.82	-	0.46 ± 1.3	-	-	0.4 ± 01.2	-	-	0.87 ± 0.78	0.54 ± 0.44	-	0.76 ± 0.98	0.45 ± 1.2	-	1.76 ± 0.78	0.72 ± 0.98	-
5	-	-	-	-	-	-	-	-	-	0.51 ± 1.2	-	-	0.51 ± 0.2	-	-	0.79 ± 1.2	0.54 ± 0.23	-
2.5	-	-	-	-	-	-	-	-	-	-	-	-	-	-	-	-	-	-
AgNO_3_	-	-	-	-	-	-	-	-	-	-	-	-	-	-	-	-	-	-
Negative Control (Distilled H_2_O)	-	-	-	-	-	-	-	-	-	-	-	-	-	-	-	-	-	-
Positive Control (Streptomycin)	2.1			2.42			2.4			3.4			4.72			4.2		

Abbreviations: AgNPs—silver nanoparticles, AgMPs—silver microparticles, conc—concentration, S.E—standard error, M.A—maleic acid, C.A—citric acid, C—uncapped control, *M. luteus*—*Micrococcus luteus, S. aureus*—*Staphylococcus aureus, B. subtilis*—*Bacillus subtiles, A. tumefaciens*—*Agrobacterium tumefaciens, S. Setubal*—*Salmonella Setubal, E. aerogenes*—*Enterobacter aerogenes.*

**Table 2:** Analysis of variance for factors affecting the viability of brine shrimp.

Source of Variation	Df	Sum-of-Squares	Mean Square	F-Value	*p*-Value	Significant
Interaction	9	3091	343.5	117.4	<0.0001	Yes
Types of particles	3	14280	4761	1628	<0.0001	Yes
Concentration	3	6649	2216	757.7	<0.0001	Yes
Residual	32	93.60	2.925			

**Table 3:** Analysis of variance for factors affecting the viability of MCF7 Cells.

Source of Variation	Df	Sum-of-Squares	Mean Square	F-Value	*p*-Value	Significant
Interaction	12	2227	185.6	97.86	<0.0001	Yes
Types of particles	3	22690	7562	3988	<0.0001	Yes
Concentration	4	5420	1355	714.6	<0.0001	Yes
Residual	40	75.84	1.896			

**Table 4:** Analysis of variance for factors affecting the viability of HePG2 Cells.

Source of Variation	Df	Sum-of-Squares	Mean Square	F-Value	*p*-Value	Significant
Interaction	12	1676	139.6	100.5	<0.0001	Yes
Types of particles	3	10340	3447	2852	<0.0001	Yes
Concentration	4	1571	392.7	374.5	<0.0001	yes
Residual	40	205.3	5.133			

**Table 5:** Analysis of variance for factors affecting the free radical scavenging activity of silver particles.

Source of Variation	Df	Sum-of-Squares	Mean Square	F-Value	p-Value	Significant
Interaction	6	1339	223.2	21.02	<0.0001	Yes
Types of particles	3	27620	9206	866.8	<0.0001	Yes
Concentration	2	3197	1599	150.5	<0.0001	Yes
Residual	24	254.9	10.62			

## Supplementary Materials

The following are available online at https://www.mdpi.com/2079-4983/11/4/76/s1 Figure S1: Scanning electron microscope (SEM) image showing target (A) of energy dispersive X-Ray spectroscopy (EDS) spectra of uncapped silver microparticles (AgMPs) with (B).Figure S2: Scanning electron microscope (SEM) image showing target (A) of energy dispersive X-Ray spectroscopy (EDS) spectra of maleic acid capped silver nanoparticles (AgNPs) (B). Figure S3: Scanning electron microscope (SEM) image showing target (A) of energy dispersive X-Ray spectroscopy (EDS) spectra of citric acid capped silver microparticles (AgMPs) (B).
