# Supplementary material for: Synthesis of Functional Silver Nanoparticles and Microparticles with Modifiers and Evaluation of Their Antimicrobial, Anticancer, and Antioxidant Activity
Source: J Funct Biomater. 2020 Oct 23;11(4):76. doi: 10.3390/jfb11040076 (PMC7711460; doi:10.3390/jfb11040076)
Supplement: Supplementary file 1 [file jfb-11-00076-s001.pdf]

Journal of
Functional
Biomaterials

MDPI

Supplementary Material

Synthesis of Functional Silver Nanoparticles and Microparticles with Modifiers and Evaluation of Their Antimicrobial, Anticancer, and Antioxidant Activity

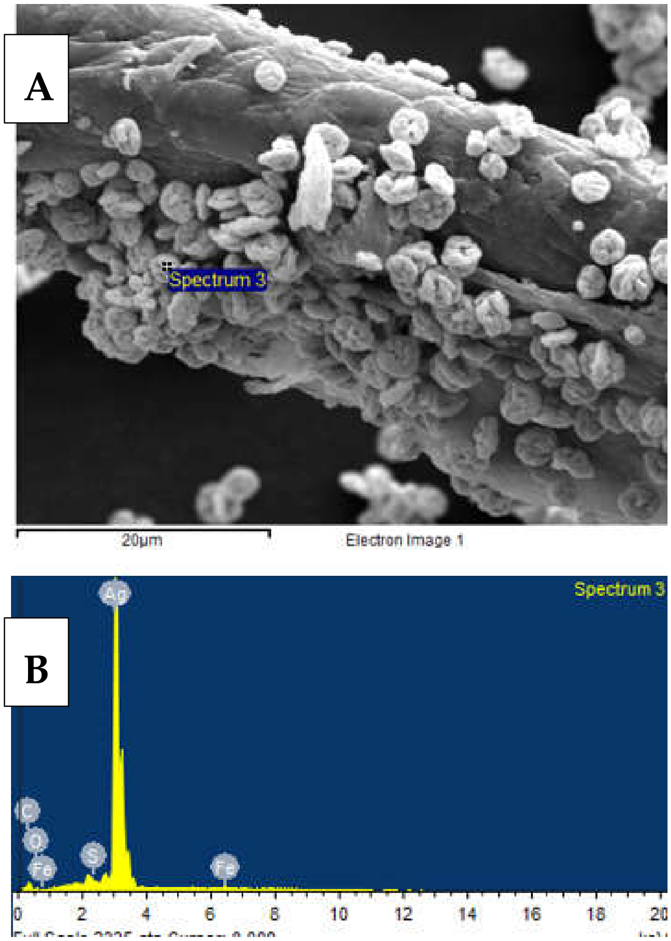

Figure S1. Scanning electron microscope (SEM) image showing target (**A**) of energy dispersive X-Ray spectroscopy (EDS) spectra of uncapped silver microparticles (AgMPs) with (**B**).

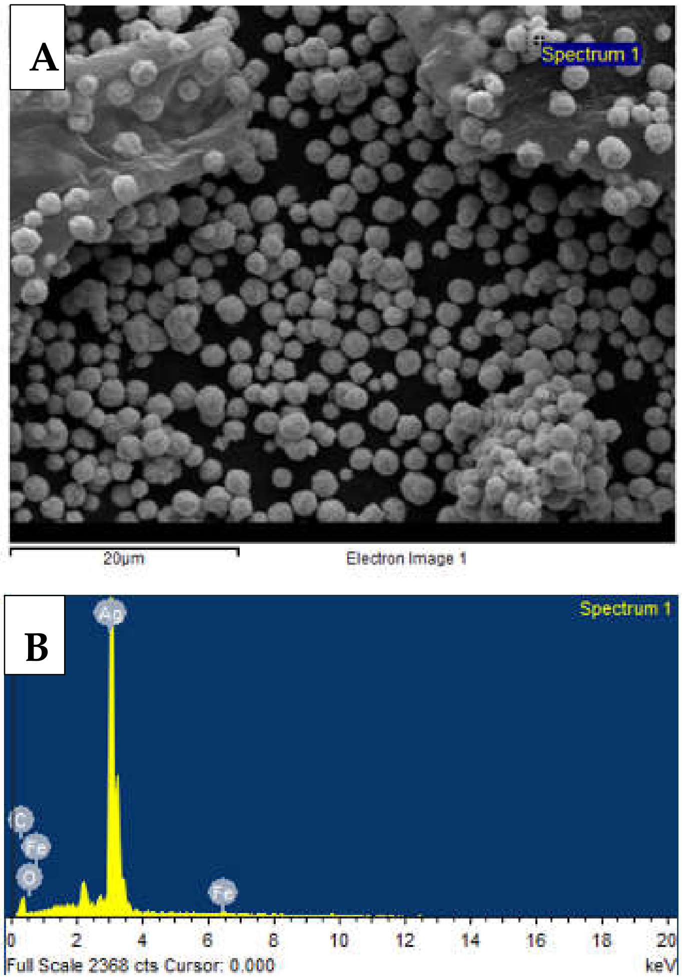

Figure S2. Scanning electron microscope (SEM) image showing target (**A**) of energy dispersive X-Ray spectroscopy (EDS) spectra of maleic acid capped silver nanoparticles (AgNPs) (**B**).

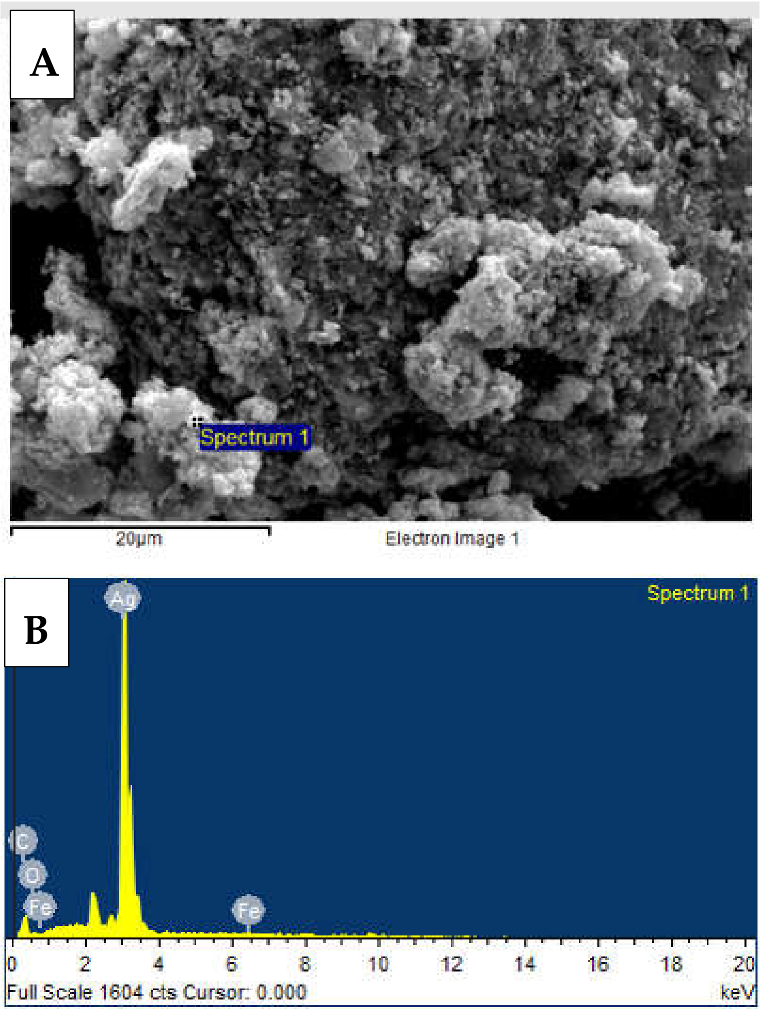

Figure S3. Scanning electron microscope (SEM) image showing target (**A**) of energy dispersive X-Ray spectroscopy (EDS) spectra of citric acid capped silver microparticles (AgMPs) (**B**).
